# Diabetes in Pakistan: A systematic review and meta-analysis

**DOI:** 10.12669/pjms.35.4.194

**Published:** 2019

**Authors:** Sohail Akhtar, Jamal Abdul Nasir, Tahir Abbas, Aqsa Sarwar

**Affiliations:** 1 Dr. Sohail Akhtar, PhD. Department of Statistics, Government College University Lahore, Lahore, Pakistan; 2 Dr. Jamal Abdul Nasir, PhD. Department of Statistics, Government College University Lahore, Lahore, Pakistan; 3 Dr. Tahir Abbas, PhD. Department of Statistics, Government College University Lahore, Lahore, Pakistan; 4 Aqsa Sarwar, BS Student. Department of Statistics, Government College University Lahore, Lahore, Pakistan

**Keywords:** Diabetes, Prediabetes, Pakistan, Meta-analysis and Systematic review

## Abstract

**Objective:**

The purpose of this study was assess the time trend of the prevalence of prediabetes and diabetes and risk factors associated with diabetes in Pakistan by using a systematic review and meta–analysis.

**Methods:**

A systematic literature search of Embase, PubMed, and the Cochrane library was carried out between January 1, 1995 and August 30, 2018. Diabetes and prediabetes prevalence estimates were combined by the random–effects model. The existence of publication bias was tested by Egger regression. This systematic review was reported following the PRISMA guidelines.

**Results:**

The search conceded a total of 635 studies, only 14 studies were considered for meta-analysis. The prevalence of diabetes in Pakistan was revealed 14.62% (10.651%–19.094%; 14 studies) based on 49,418 people using the inverse–variance random–effects model. The prevalence of prediabetes was 11.43% (8.26%–15.03%; 10 studies) based on a total sample of 26,999 people. The risk factors associated with diabetes were mean age (β = 0.48%, 95% CI: 0.21–0.78, p<0.001), the proportion of participants with a family history of diabetes (β = 0. 45%, 95% CI: 0.08–0.82, p =0.018, p<0.001), hypertension (β = 0.40%, 95% CI: 0.06–0.75, p = 0.022), weight (BMI) (β = 0.21%, 95% CI: 0.02–0.4, p=0.030).

**Conclusions:**

There has been a continuous increase in the prevalence of prediabetes and diabetes in Pakistan. All parts of the country have been affected, with the highest in Sindh and lowest in Khyber Pakhtunkhwa. The main factors include growing age, family history, hypertension and obesity. A nationwide diabetes care survey on risk factors and prevention policy is highly recommended.

## INTRODUCTION

Diabetes is one of the fastest rising public health issues and causing a number of serious health complications. The prevalence of diabetes is growing globally due to aging factor, physical inactivity, overweight, urbanization, sedentary lifestyle and poor eating habits.^1^ Globally, it has been projected that the number of diabetes people will be rising to 693 million by 2045 from 451 million in 2017.^2^ It is also estimated that 49.7% of people living with type-II diabetes are undiagnosed.^3^ In the patients with type-II diabetes, the average life expectancy is decreased by around 10 years.^4^ In the developing countries, majority of diabetes patients are under 64 years of age, while in developing countries, most are in higher age groups.^2^ Diabetes in adult population is expected by 69 percent from 2010 to 2030 in the developing countries as compared to 20 percent for developed countries.^4^,^5^ Pakistan is a developing country and facing a sharp growth in the prevalence of diabetes. Although, several research studies have been performed to investigate the prevalence of diabetes and its associated risk factors, but estimates of the prevalence of diabetes vary widely from study to study. There are no solid and consistent prevalence data are available to find the trends over time period. The purpose of this study was to summarize current data to find out the trends and pooled prevalence of diabetes, prediabetes and undiagnosed diabetes in a general adult population living in Pakistan. Furthermore, we also analyzed the correlated risk factors of diabetes.

## METHODS

### Search Strategy

We systematically searched articles on PubMed, Medline, EMBASE, the Cochrane Library, and Pakistani Journals Online websites [for example: Journal of Pakistan Medical Association (www.jpma.org.pk/); Journals of the College of Physicians and Surgeons Pakistan (www.jcpsp.pk); Pakistan Journal of Medical Sciences (www.pjms.org.pk), etc] from January 1995 to August 2018. Using MeSH headings, the terms ‘‘diabetes mellitus,’’ “prediabetes”, “Impaired glucose tolerance (IGT)”, ‘‘risk factors’’, ‘‘prevalence,’’ “glucose abnormalities”, “glucose intolerance” and ‘‘Pakistan” as well as variations thereof were searched for. Results were described using the Preferred Reporting Items for Systematic and Meta-analyses (PRISMA) guidelines (Table-I).^6^

**Table I:** Characteristics of 14 studies included in the analysis.

	Author	Year of publication	Period of inclusion	Sampling Method	Setting	Province	% Response rate	% Female	% Male	% Overweight	% Obesity	% Hypertension	% Positive Family History	% Low Physical Activity
15	Shera et al.	1995	Feb to Mar 1994	Random Sampling	Ruler	Sindh	76.40	NA	NA	62.23	NA	49.39	43.06	NA
16	Shera et al.	1999	NA	Random Sampling	Ruler	KP	NA	80	20	49.28	NA	21.22	16.78	NA
17	Basit et al.	2002	NA	Random Sampling	Ruler	Baluchistan	NA	67.02	33	NA	38.3	20.27	0.88	NA
18	Basit et al.	2011	Feb 2009 to Feb 2010	Random Sampling	Ruler	Baluchistan	NA	NA	NA	NA	NA	54.27	32.75	NA
19	Akhtar et al.	2016	NA	Random Sampling	Ruler	KP	NA	49.20	50.80	NA	NA	Na	Na	NA
20	Rifat	2009	Feb 1,2007 to Jan 31,2008	Random Sampling	Urban	Punjab	NA	49.30	50.78	17.92	26.08	NA	22.46	NA
21	Zafar et al.	2010	Jul-08	Random Sampling	Urban	Punjab	NA	73.14	26.86	14.5	16.17	NA	NA	15.89
22	Sohail	2014	Jan 2006 to Dec 2008	Random Sampling	Urban	Sindh	NA	NA	NA	NA	NA	NA	NA	NA
23	Zafar et al.	2016	May to Sep 2014	Random Sampling	Urban	Punjab	NA	55.20	44.80	34.4	35.4	50.3	43.34	29.5
24	Ahmad et al.	2017	Jan to Aug 2016	Random Sampling	Urban	Punjab	NA	80.65	19.35	33	42	NA	43	NA
25	Shera et al.	1999	Mar to Jun 1995	Random Sampling	Both	Baluchistan	77.50	69.00	30.90	38.28	NA	14.03	32.95	NA
26	Shera et al.	2007	NA	Randomized and cluster sampling	Both	All 4 Provinces	NA	65.15	34.84	NA	NA	42.45	26.08	NA
27	Shera et al.	2010	NA	Random Sampling	Both	Punjab	50.72	56.05	44.01	32.46	NA	47.57	24.15	NA
28	Basit et al.	2018	Feb 2016 to Mar 2018	Multistage Stratified Sampling	Both	All 4 Provinces	87	56.10	43.90	NA	NA	47.4	30.2	NA

### Inclusion and exclusion criteria

Only population based studies that were carried out between January 1995 and August 2018 were considered in the meta-analysis. Hospital-based and clinical studies were excluded from the meta-analysis. Pakistani community living outside Pakistan, or those studies considered pregnant women or children were excluded from the analysis.

### Data Extraction

Different information was extracted from the qualified studies, such as first author name, year of publication, gender, age, studied sample, the prevalence rate of diabetes and prediabetes, smoking, survey year, study setting (urban, rural or both) study design, sampling method, and geographic region (province) in which the study was carried out. An extract of the data is presented Table-I.

### Statistical Analyses

The prevalence of diabetes and prediabetes were examined and analyzed using the software R version. 3.5.1.^7^ for Microsoft Windows, using two packages meta 4.9-2 and metafor 2.0. Random effect meta-analysis models were used to find out the pooled prevalence for diabetes, prediabetes and undiagnosed diabetes. Because of the considerable heterogeneity observed between individual studies, a random-effects meta-analysis was used to adjust for variability and pool the study specific prevalence rates.^8^,^9^ To stabilize the variance of each study, we used Freeman Tukey Double Arcsine transformation.^10^ For quantifying statistical Heterogeneity across studies, Cochrane’s Q-statistic,^11^ and I²-Statistic were used.^12^ Heterogeneity was categorized as high, moderate, low and, with *I*^2^ value 75%, 50% and 25% respectively. To investigate possible reasons of heterogeneity, meta-regression and subgroup analyses were used by areas, year of publication, gender, and age. The existence of publication bias was initially checked by the graphical display of funnel plot and then test by the Egger’s.^13^,^14^

### Literature Search

**Fig. 1:**
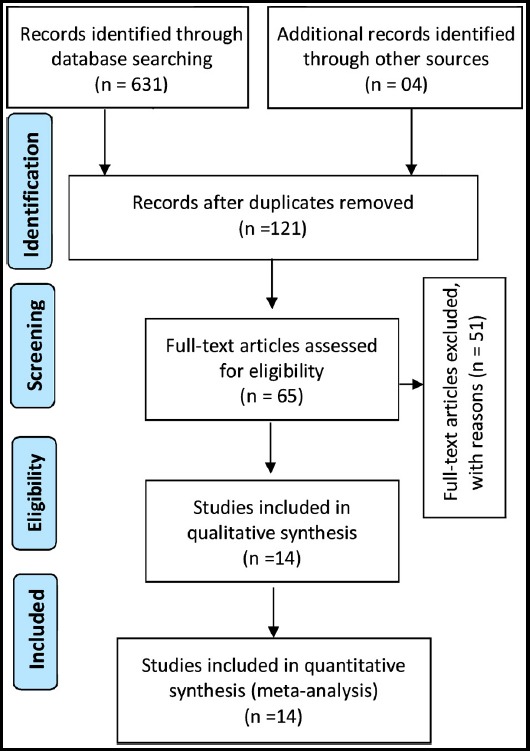
Flow diagram explaining the number of included and excluded articles in the meta-analysis on diabetes in Pakistan, considered from the PRISMA 2009 guideline.^6^

The literature search yielded 635 articles eligible for analysis. Five hundred and fourteen duplicated studies were removed. After reviewing titles and abstracts, 56 articles were found irrelevant and then excluded from the process. As a result, only 65 studies were selected for full-text reading. Later, 56 articles were excluded after full text read for the following reasons: articles with no numerical prevalence measure(s) of diabetes; studies that were not based in Pakistan; studies with no clear assessment methods or grading systems of diabetes; studies based on hospital data set or eligibility criteria not met or full-text did not include relevant indicators. Finally, only 14 articles met the inclusion criteria and data were extracted for the analysis. The flow chart of study selection process is presented in Fig.1, considered from the PRISMA flow diagram.^6^

### Methodological quality and characteristics of included studies

All studies were cross-sectional. The simple random sampling procedure was used 12 out of 14 studies. The articles were published between 1995 and 2018 while the period of subject inclusion was from Feb. 1995 to Mar. 2017. Diabetes was reported based on the self-reporting (known diabetes) and different diagnostic tests: A1C criteria, fasting plasma glucose (FPG) and 2-h plasma glucose (2-h PG). All the four provinces of Pakistan were represented in articles. Five studies were conducted in a rural regions15-19 while five in an urban region20-24 and four in both regions.25-28 The proportion of females ranged from 49.20% to 81.65%. The mean age varied from 18 to 76 years (14 studies).15-28 The proportion of hypertension ranged from 14.4% to 43.43%.16-18,23,25-28 The proportion of people who had positive family history varied from 0.88% to 43.3% (11 studies).15-18 20 23-28 Obesity ranged from 16.16% to 42% (5 studies).16,17,19,20,22, The proportion of people with overweight body mass index ranged from 17.93% to 62.23% (8 studies).15,16, 20-24,27 The statistics of the included studies were presented in Table-I.

## RESULTS

**Fig. 2:**
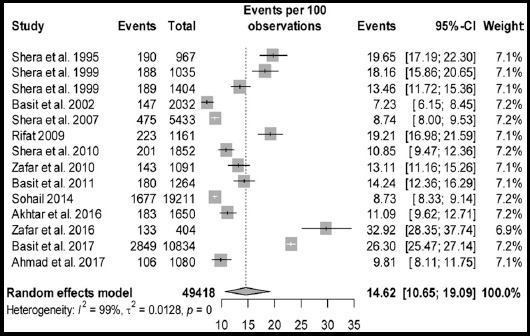
Forest plot of prevalence of diabetes from population Jan. 1995 to Aug. 2018.

**Fig. 3:**
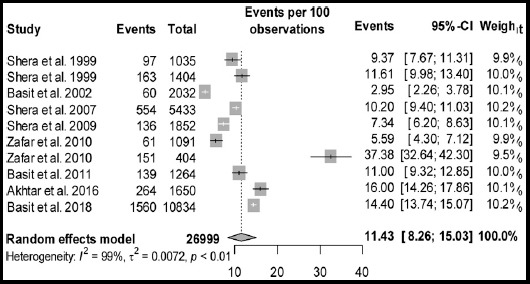
Forest plot of prevalence of prediabetes from population Jan. 1995 to Aug. 2018.

**Fig. 4:**
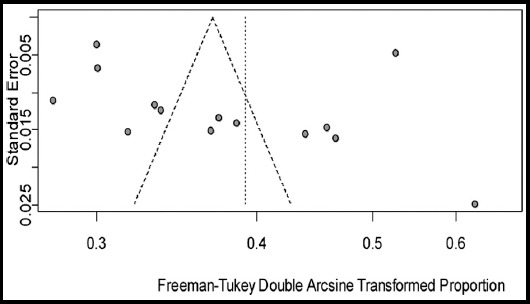
Funnel plot of the prevalence of diabetes in Pakistan from Jan. 1995 to Aug. 2018.

Statistical analyses of prevalence of diabetes and prediabetes are presented in Table-II. The pool prevalence of diabetes was 14.62% (95% CI: 10.651-19.09, I² = 99.3%, 14 studies) in a total sample of 49,418 participants (Fig.2). The funnel plot (Fig.3) showed publication bias which is confirmed by the Egger’s test (p = 0.656). The prevalence of prediabetes was 11.43 % (95%CI: 8.26-15.03, I² = 98.50%, 10 studies) in a total sample size of 26,999 individuals. The forest plot of prediabetes in presented in Fig.4. The prevalence of undiagnosed diabetes was 9.27% (95% CI: 3.25-17.94), I² = 99.70%, 6 studies) in a total sample size of 36,748 individuals.

**Table II:** Prevalence of diabetes, prediabetes and its risk factors in the adult population of Pakistan, from Jan. 1995 to Aug. 2018.

Column1	Studies	Sample	Cases	Prevalence, % (95%CI)	I², %	Heterogeneity	P-Egger test
Diabetes	14	49418	6884	14.62(10.651-19.09)	0.993	< 0.001	0.6559
Undiagnosed	6	36748	1443	9.27(3.25-17.94)	0.997	< 0.001	0.1267
Prediabetes	10	26999	3185	11.43(8.26-15.03)	0.985	< 0.001	0.6508
By Sex							0.0278
Male	10	6131	817	14.80(9.83-20.59)	0.982	< 0.0001	
Female	10	11011	1811	15.83(10.05-22.63)	0.976	< 0.0001	
By setting							0.374
Urban	5	5472	845	17.72(12.22-23.98)	0.969	< 0.001	
Ruler	7	10969	1206	12.10(8.75-15.89)	0.969	< 0.001	
By Age	5						
25-34	5	3119	93	3.24(2.32-4.30)	0.5	0.0915	0.0044
35-44	5	2544	275	12.83(8.43-17.97)	0.909	< 0.001	
45-54	5	2212	365	19.52(13.56-26.25)	0.918	< 0.001	
55-64	5	1642	288	20.73(14.69-27.50)	0.886	< 0.001	
65-74	5	855	160	21.84 (15.36-30.08)	0.817	0.0002	
75+	5	319	60	18.86 (8.16-37.81)	0.871	< 0.001	
By Province							0.2263
Panjab	6	11809	2685	18.52(10.74-27.82)	0.992	< 0.001	
Sindh	3	22709	2683	19.25(5.60-38.48)	0.998	< 0.001	
Baluchistan	4	5238	675	15.25(8.56-23.43)	0.982	< 0.001	
Khyber Pakhtunkhwa	3	4229	575	13.98(10.39-18.00)	0.923	< 0.001	

### Source of heterogeneity and subgroup analysis

In Table-II, subgroup analysis stratified by gender—prevalence among females were revealed to be 15.83% higher than males 14.80% (10.05%-22.63%) females, while in male 14.80% (9.83%-20.59%). Pooled prevalence of age-groups in 25-34 yrs, 35-44 yrs, 45-54 yrs and 55-64 yrs, 65-74 yrs and 75+ yrs were 3.24% (2.32%-4.30%), 12.83% (8.43%-17.97%), 19.52% (13.56%-26.25%), 20.73% (14.69%-27.50%), 21.84% (15.36%-30.08%), and 21.84% (15.36%-30.08%), respectively. The prevalence in the 65-74 years age-group was the highest of the six age groups, and the prevalence of diabetes increased with age gradually. With regard to province studies, the prevalence of diabetes was high 19.25% (5.60%-38.48%) of Sindh, compared with 18.52% (10.74%-27.82%) of Punjab, 15.25% (8.56%-23.43%) of Baluchistan and 13.98% (10.39%-18.00%) of Khyber Pakhtunkhwa.

The subgroup analysis of diabetes is presented in Table-II. The prevalence of diabetes increases with the growing age. The prevalence of diabetes between male and female was insignificant and between urban and rural regions. There was no significant publication bias for all subgroup analyses. Using the univariate meta regression analysis, the prevalence of diabetes increased sharply with age (*β* = 0.49%, 95% CI: 0.21-0.78, p<0.001 with *R*^2^= 75.63), the proportion of participants with hypertension (*β* = 0.40%, 95% CI: 0.06-0.75, p = 0.022, *R*^2^=40.80), the proportion of participants with a family history of diabetes (*β* = 0. 45%, 95% CI: 0.08-0.82, p=0.018, *R*^2^= 30.35) and BMI (*β* = 0.21%, 95% CI: 0.02-0.4, p =0.0295, *R*^2^= 21.32). The prevalence of diabetes was not correlated with smoking at the time of data collection, inclusion time period, physical inactivity and waist hip ratio obesity.

## DISCUSSION

To the best of our knowledge, this is the first study to determine the prevalence of and risk factors for diabetes in Pakistan using a systematic review and meta-analysis. The pooled prevalence of diabetes was revealed 14.62% (based on 49,418 individuals) which suggest that there has been a significant increase in the prevalence of diabetes in Pakistan. Furthermore, the selected studies in this meta-analysis cover almost all geopolitical zones of Pakistan, making it possible to determine regional differences in the prevalence of Diabetes. Diabetes is affecting all around the country, with the highest prevalence seen in the Sindh province and with the lowest in Khyber Pakhtunkhwa. Growing age, family history, hypertension, overweight, are important risk factors for diabetes among Pakistanis. A nationwide diabetes care survey and prevention policy is highly recommended.

### Limitations of the study

Out of fourteen selected studies, only two surveys reported countrywide prevalence. Secondly, the fact that we selected studies which used different screening methods for the diagnosis of diabetes means that some people with the disease could have been missed. Furthermore, significant heterogeneity was found in combining the prevalence rates of diabetes. The main sources of heterogeneity in the included studies related to the different characteristics of study population.

### Authors’ Contribution:

**SA, JAN & TA:** Conceived, designed and did statistical analysis & editing of manuscript.

**SA, AS & TA:** Did data collection and manuscript writing.

**SA, JAN & AS:** Did review and final approval of manuscript.
